# Geographical distribution and genetic diversity of *Plasmodium vivax* reticulocyte binding protein 1a correlates with patient antigenicity

**DOI:** 10.1371/journal.pntd.0010492

**Published:** 2022-06-23

**Authors:** Ji-Hoon Park, Min-Hee Kim, Edwin Sutanto, Seok-Won Na, Min-Jae Kim, Joon Sup Yeom, Myat Htut Nyunt, Mohammed Mohieldien Abbas Elfaki, Muzamil Mahdi Abdel Hamid, Seok Ho Cha, Sisay Getachew Alemu, Kanlaya Sriprawat, Nicholas M. Anstey, Matthew J. Grigg, Bridget E. Barber, Timothy William, Qi Gao, Yaobao Liu, Richard D. Pearson, Ric N. Price, Francois Nosten, Sung-Il Yoon, Joo Hwan No, Eun-Taek Han, Sarah Auburn, Bruce Russell, Jin-Hee Han

**Affiliations:** 1 Department of Medical Environmental Biology and Tropical Medicine, School of Medicine, Kangwon National University, Chuncheon, Republic of Korea; 2 Eijkman Institute for Molecular Biology, Jakarta, Indonesia; 3 Department of Infectious Diseases, Asan Medical Center, Seoul, Republic of Korea; 4 Department of Internal Medicine, Yonsei University College of Medicine, Seoul, Republic of Korea; 5 Department of Medical Research, Yangon, Myanmar; 6 Department of Parasitology and Medical Entomology, Institute of Endemic Diseases, University of Khartoum, Khartoum, Sudan; 7 Department of Microbiology and Parasitology, Faculty of Medicine, Jazan University, Jizan, Saudi Arabia; 8 Department of Parasitology and Tropical Medicine, Inha University School of Medicine, Incheon, Republic of Korea; 9 College of Natural Sciences, Addis Ababa University, Addis Ababa, Ethiopia; 10 Armauer Hansen Research Institute, Jimma Road, Addis Ababa, Ethiopia; 11 Bioreliance, Rockville, Maryland, United States of America; 12 Shoklo Malaria Research Unit, Mahidol-Oxford Tropical Medicine Research Unit, Faculty of Tropical Medicine, Mahidol University, Mae Sot, Tak, Thailand; 13 Global and Tropical Health Division, Menzies School of Health Research and Charles Darwin University, Darwin, Australia; 14 Infectious Diseases Society Sabah-Menzies School of Health Research Clinical Research Unit, Sabah, Malaysia; 15 Clinical Research Centre, Queen Elizabeth Hospital, Sabah, Malaysia; 16 Gleneagles Hospital, Sabah, Malaysia; 17 National Health Commission Key Laboratory of Parasitic Disease Control and Prevention, Jiangsu Provincial Key Laboratory on Parasite and Vector Control Technology, Jiangsu Institute of Parasitic Diseases, Wuxi, China; 18 School of Public Health, Nanjing Medical University, Nanjing, China; 19 Wellcome Sanger Institute, Hinxton, Cambridge, United Kingdom; 20 Centre for Tropical Medicine and Global Health, Nuffield Department of Clinical Medicine Research Building, University of Oxford, Oxford, United Kingdom; 21 Mahidol-Oxford Tropical Medicine Research Unit, Faculty of Tropical Medicine, Mahidol University, Bangkok, Thailand; 22 Division of Biomedical Convergence, College of Biomedical Science, Kangwon National University, Chuncheon, Republic of Korea; 23 Host-Parasite Research Laboratory, Institut Pasteur Korea, Seongnam, Republic of Korea; 24 Department of Microbiology and Immunology, University of Otago, Dunedin, New Zealand; Temple University, UNITED STATES

## Abstract

*Plasmodium vivax* is the most widespread cause of human malaria. Recent reports of drug resistant vivax malaria and the challenge of eradicating the dormant liver forms increase the importance of vaccine development against this relapsing disease. *P*. *vivax* reticulocyte binding protein 1a (PvRBP1a) is a potential vaccine candidate, which is involved in red cell tropism, a crucial step in the merozoite invasion of host reticulocytes. As part of the initial evaluation of the PvRBP1a vaccine candidate, we investigated its genetic diversity and antigenicity using geographically diverse clinical isolates. We analysed *pvrbp1a* genetic polymorphisms using 202 vivax clinical isolates from six countries. *Pvrbp1a* was separated into six regions based on specific domain features, sequence conserved/polymorphic regions, and the reticulocyte binding like (RBL) domains. In the fragmented gene sequence analysis, PvRBP1a region II (RII) and RIII (head and tail structure homolog, 152–625 aa.) showed extensive polymorphism caused by random point mutations. The haplotype network of these polymorphic regions was classified into three clusters that converged to independent populations. Antigenicity screening was performed using recombinant proteins PvRBP1a-N (157–560 aa.) and PvRBP1a-C (606–962 aa.), which contained head and tail structure region and sequence conserved region, respectively. Sensitivity against PvRBP1a-N (46.7%) was higher than PvRBP1a-C (17.8%). PvRBP1a-N was reported as a reticulocyte binding domain and this study identified a linear epitope with moderate antigenicity, thus an attractive domain for merozoite invasion-blocking vaccine development. However, our study highlights that a global PvRBP1a-based vaccine design needs to overcome several difficulties due to three distinct genotypes and low antigenicity levels.

## Author summary

*Plasmodium vivax* shows restricted host cell tropism to human reticulocytes for their asexual blood-stage replication. Reticulocyte binding protein (PvRBPs) family are essential molecules for reticulocyte recognition and host cell invasion. In this study, we focused on PvRBP1a as a novel target for vaccine development of *P*. *vivax*. Here, we analysed *pvrbp1a* genetic polymorphisms, natural selection, and evaluated the correlation of this genetic information with antigenicity. This study emphasizes the characteristics of the *pvrbp1a* genetic population in relation to efficient vaccine development.

## Introduction

In 2020 the prevalence of *Plasmodium vivax* malaria was estimated to be between 4.1 and 5.1 million cases globally [1,2], accounting for a significant proportion of malaria cases in the African (0.3%), South-East Asia (36.3%), and Western Pacific (30.1%) [1]. Despite its global importance, *P*. *vivax* research has been neglected compared to malaria caused by *P*. *falciparum* due in part to its low directly associated mortality and the absence of a continuous *in vitro* culture method (a consequence of *P*. *vivax* only invading nascent CD71+ve reticulocytes). As most malaria elimination programs target *P*. *falciparum*, the proportion of *P*. *vivax* infections outside of sub-Saharan Africa continues to increase [3,4]. Failure to control *P*. *vivax* is primarily due to relapsing blood infections, emerging from activated dormant liver forms (hypnozoites) often weeks to months after the primary infection. This is compounded by a cryptic endosplenic life-cycle resulting in a persistent hidden splenic reservoir of asexual-stage *P*. *vivax* parasites in chronic infection [5,6]. Additionally, increasing reports of drug resistant vivax malaria in South-East Asia [7], have spurred efforts to develop a vaccine against *P*. *vivax*.

Most vaccine efforts against *P*. *vivax* have focused on the assumption that its erythrocytic life cycle is dependent on the presence of the Duffy Antigen Receptor for Chemokine (DARC, CD234) on the host red cell. Therefore, targeting DARC’s corresponding ligand (*P*. *vivax* Duffy Binding Protein, PvDBP) is the most investigated *P*. *vivax* vaccine target. Of particular interest is the extracellular region II of the PvDBP (PvDBP-RII) which specifically interacts with the Fy6 region of DARC [8]. Although PvDBP is a promising vaccine candidate, there are several challenges. Firstly, a general lack of understanding of effective PvDBP-RII vaccine conditions such as the method of delivery, immune-reactivity, and persistence [9,10]. Secondly, a failure of the PvDBP-RII vaccine in field trials resulting from high levels of natural genetic polymorphisms [11–13]. Finally, the widespread discovery of Duffy-negative vivax malaria cases in Madagascar and parts of Northern Africa, suggesting *P*. *vivax* can use alternate invasion pathways besides DARC [14]. *P*. *vivax* reticulocyte binding-like protein family (PvRBL) is another promising vaccine candidate [15], consisting of eleven members including three pseudogenes [16]. Although their precise roles remain largely unknown, the RBL protein family such as PvRBP2a, PvRBP2b, and *P*. *falciparum* reticulocyte binding-like protein homologs (PfRh) show consistent functions of host erythrocyte binding activity (*i*.*e*. enabling *P*. *vivax* merozoites to identify host reticulocytes) [17,18]. The most well-known member of RBL protein family in *P*. *vivax* is PvRBP2b. This RBL protein interacts with the transferrin receptor (CD71), which is an important marker for selecting young reticulocyte [19]. Additionally, monoclonal antibodies against PvRBP2b showed inhibition of *P*. *vivax* invasion in Brazilian and Thai clinical isolates [19]. Another PvRBL family member, PvRBP1a is also shown to bind preferentially to human reticulocytes [20–24] with affinity-purified patient IgG against PvRBP1a fragment (30–778 aa. and 352–599 aa.) specifically blocking reticulocyte binding of native and recombinant PvRBP1a antigens [21,22]. These studies support the use of PvRBL proteins as attractive vaccine candidates for targeting the alternative *P*. *vivax* invasion pathways.

In this study, we analysed clinical isolates from six countries for genetic diversity and three countries for the antigenicity screening of PvRBP1a. Comparing vivax malaria patient antigenicity, genetic polymorphism, and natural selection we aimed to produce new information on the suitability of PvRBP1a as a vaccine candidate.

## Methods

### Ethics statement

Whole blood samples were collected from symptomatic *P*. *vivax* patients after microscopic blood film examination at regional health centres of the respective countries. All clinical samples were collected under the following ethical guidelines and approved protocols: Kangwon National University Hospital Ethical Committee, Republic of Korea (Ref. No. 2014-08-008-002), Department of Medical Research, Republic of the Union of Myanmar (Approval No-52/Ethics, 2012), Human Research Ethics Committee of Northern Territory Department of Health and Families (HREC-13-1942, HREC-2010-1431, HREC-2012-1815 and HREC-2010-1396), the Medical Research Ethics Committee, Ministry of Health, Malaysia (NMMR-10-754-6684, NMRR-12-511-12579), Institutional Review Board of Jiangsu Institute of Parasitic Diseases, Wuxi, China (IRB00004221), Mahidol University Faculty of Medical Technology, Thailand (MUTM 2011-043-03), Addis Ababa University College of Natural Sciences, Ethiopia (RERC/002/05/2013), Armauer Hansen Research Institute, Addis Ababa, Ethiopia (AHRI-ALERT P011/10) and National Research Ethics Review Committee of Ethiopia (Ref. No. 3.10/580/06), the Scientific Research and Ethics Committee of the Institute of Endemic Diseases, University of Khartoum, Sudan (Ref. No. 9/2016). All adult participants provided informed written consent, and a parent or guardian of any child participant provided informed written consent on their behalf.

### Genomic DNA extraction, sequencing, and sequence data collection

Genomic DNA was extracted from 200 μL whole patient blood samples using Genomic DNA Extraction Kit (Bioneer, ROK) following the manufacturer’s protocol. In total, 26 isolates from ROK and Myanmar were amplified (Republic of Korea (ROK, *n* = 16) and Shwegyin in Myanmar (*n* = 10)). For the whole *pvrbp1a* gene sequencing, a total of 9 primer sets was designed based on *pvrbp1a* (PVP01_0701200) derived from the PvP01 Papua Indonesian reference sequence (S1 Table) [25]. The detailed whole gene sequences of *pvrbp1a* are available at GenBank (accession numbers: MW862435—MW862460).

*pvrbp1a* sequences from China (*n* = 4), Ethiopia (*n* = 22), Malaysia (Sabah, *n* = 54), and Thailand (North-western, n = 96) were obtained from whole-genome sequencing data which were described previously at [26–29]. Briefly, the genomic region for *pvrbp1a* was obtained from PlasmoDB [30]. Reads that were aligned to this region were extracted from the whole-genome sequencing data (in BAM format) as FASTQ files using samtools [31]. For each sample, a de novo assembly was performed to the available reads using SPAdes [32]. From the SPAdes output, the longest contig from each sample was collected into a single FASTA file. The reference sequence for *pvrbp1a* was added into this FASTA file for multiple sequence alignment using MAFFT [33]. Samples that did not assemble correctly (i.e., structurally deviated from the reference sequence) were removed. After removal, there were sequences that had indels compared to the *pvrbp1a* reference. To validate the presence of these indels, the Genome Analysis Toolkit (GATK) [34] was used to realign these indels to the reference sequence. Samples whose indels were not consistent between the realignment and de novo assembly results were excluded from the analysis. Finally, the remaining samples were realigned with MAFFT to generate the *pvrbp1a* sequences used in downstream analyses. A total of 202 sequences were aligned with *pvrbp1a* (PVP01_0701200) sequence.

### Homology-based tertiary structure modelling

The tertiary structure model of PvRBP1a was generated by homology-based structure prediction. Suitable structural templates were searched, and models were built using SWISS-MODEL (https://swissmodel.expasy.org) (PDB ID: 6d03.1) [35]. The quality and potential errors were evaluated by Ramachandran plots [36] and ERRAT [37]. The generated PvRBP1a initial model was refined using Galaxy Refine to improve accuracy [38]. The Root-mean-square deviation (RMSD) and Z-score were measured by DALI server (http://ekhidna2.biocenter.helsinki.fi/dali). The RMSD measures the structural differences between the aligned alpha-carbon positions. The crystallographic model of proteins with about 50% sequence identity differs by about 1 Å. The Z-score is the distance, in standard deviations, between the observed alignment RMSD and the mean RMSD for random pairs of the same length, with the same or fewer gaps. Similarities with a Z-score lower than 2 are spurious. The structure was visualized using UCSF CHIMERA software [38].

### Nucleotide diversity and natural selection

*Pvrbp1a* nucleotide diversity (*π*) is defined as the average number of nucleotide differences per site between two sequences. The number of polymorphic sites, haplotypes, and haplotype diversity (Hd) were calculated using DnaSP software [27,39]. Intra-species tests for evidence of selection (non-random evolution) were evaluated using Tajima’s D, Fu and Li’s D*, and Fu and Li’s F*. Under neutrality (random evolution), Tajima’s D is expected to be 0. Significant positive values of Tajima’s D indicate a departure from random mutation that may reflect population contraction or balancing selection, whereas significant negative values may reflect population expansion after a recent bottleneck or a recent selective sweep [40]. Significant positive values of Fu and Li’s D* and Fu and Li’s F* tend to reflect population contraction due to selection, while negative values reflect population expansion and excess of singletons [41]. Additionally, Nei and Gojobori’s method was used to calculate the intra-species (within *P*. *vivax*) proportion of synonymous substitutions per synonymous site (dS) and non-synonymous substitutions per non-synonymous site (dN) with 1,000 pseudo replication bootstraps using MEGA 7 software. A dN-dS greater than zero reflect positive selection, while a dN-dS value less than zero reflects purifying selection. Lastly, the inter-species (between *P*. *cynomolgi*) McDonald-Kreitman (MK) test, which compares the amount of variation within a species to the divergence between species. The MK test was computed using the *P*. *cynomolgi rbp1a Breok* strain (JQ422037) as an out-group species using DnaSP software.

*Pvrpb1a* haplotype diversity was evaluated using DnaSP software, and clustering patterns were assessed and illustrated using a median-joining method in Network 5.0 software. To check the robustness of the network, the haplotype tree was drawn by the maximum likelihood method and robustness was estimated by the bootstrap method with 1,000 pseudo replicates as implemented in the MEGA7.

### Recombinant protein expression

The recombinant PvRBP1a (PVP01_0701200) was expressed with two fragments for antigenicity screening. The PvRBP1a-N (157–650 aa.) was selected based on the tertiary structure homolog regions of the PvRBP2b binding domain. PvRBP1a-C (606–962 aa.) was chosen because of the sequence conserved domain within *pvrbp1a*. The recombinant PvRBP1a-N and PvRBP1a-C fragments were produced by HEK293E (HEK293 EBNA1-6E) cell-based protein expression systems. Briefly, synthesized *pvrbp1a* genes which were codon-optimized and mutated in predicted N-glycosylation sites were amplified using a specific primer set (S1 Table) and cloned into expression vector pTT5-MCS-His using In-Fusion Cloning kit (Clontech, Mountain View, CA, USA). For protein expressions, we used HEK293 EBNA1-6E cell-based transiently transfection systems. After five days of transfection, secreted proteins were collected from the culture supernatant. The expressed proteins were purified by Ni-NTA agarose (QIAGEN) with 250 mM imidazole, respectively. Each recombinant protein (3 μg/lane) was prepared with 5x reducing buffer and separated by 13% SDS-PAGE. After electrophoresis, SDS-PAGE gels were stained with Coomassie brilliant blue (Sigma-Aldrich).

### Antigenicity evaluation

Protein microarray was performed for evaluating total antigenicity using recombinant PvRBP1a-N and PvRBP1a-C. Three aminopropyl-coated slides were prepared as described previously [42]. The slides printed with optimised concentration of recombinant protein (PvRBP1a-N, 100 ng/μl and PvRBP1a-C, 50 ng/μl) to each spot were incubated for 2 hours at 37°C, and these slides were blocked with blocking buffer (5% BSA in PBS-T, 0.1% Tween 20) for 1 hour at 37°C. Healthy and vivax malaria patient sera were diluted in PBS-T to 1:25 ratio and treated on the chip for 1 hour at 37°C. The arrays were visualized with 10 ng/μl of Alexa Fluor 546 goat anti-human IgG (Invitrogen, Carlsbad, CA, USA) in PBS-T for 1 hour at 37°C and scanned using InnoScan 300 (INNOPSYS, France). The positive cut-off values calculated by negative control mean fluorescence intensity (MFI) plus two standard deviations.

Additionally, antigens were heat-treated at 80°C for 5 minutes to characterise the epitope. The linearized antigen also used for the microarray as described above.

### Statistical analysis

The antigenicity data were analysed using GraphPad Prism (GraphPad Software, San Diego, CA, USA) and SigmaPlot (Systat Software Inc., San Jose, CA, USA). For the protein array, the Student’s *t*-test was used to compare the experimentally measured values of each group. The correlation of clinical information with antigenicity was calculated by Pearson correlation test (*ρ*). Differences of *p* < 0.05 were considered significant. The total IgG reactivity index was calculated by each MFI divided by average negative MFI of PvRBP1a-N (157–650 aa.) and PvRBP1a-C (606–962 aa.), respectively, for normalization and reactivity comparison.

## Results

### *Pvrbp1a* gene sequence characteristics and tertiary structure prediction

**Fig 1:**
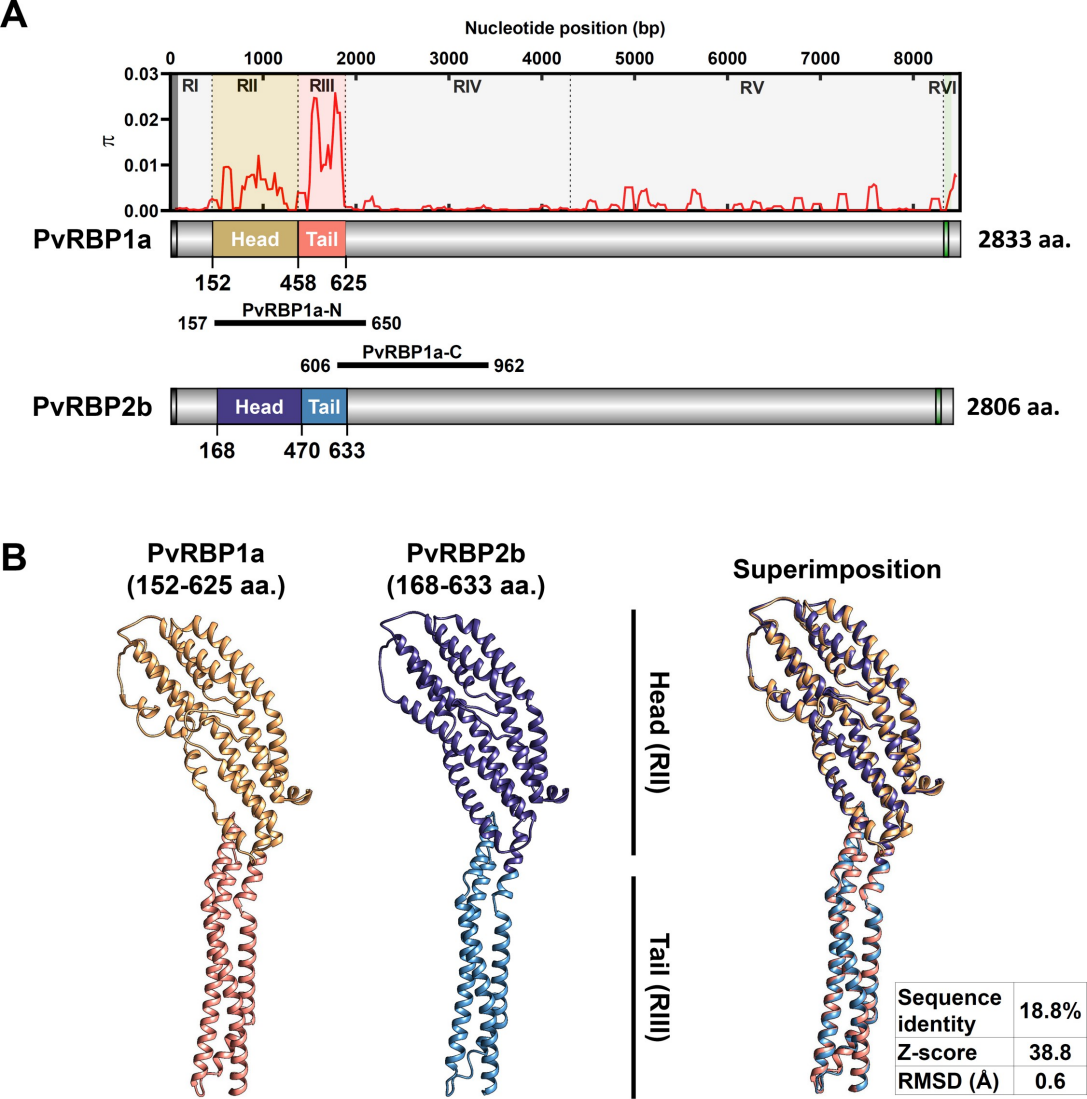
Schematic diagram of PvRBP1a and protein tertiary structure prediction. The region of PvRBP1a was divided based on specific features domain, polymorphism patterns, and structure homology between PvRBP2b and PvRBP1a. (A) PvRBP1a fragments were divided into six regions, and each fragment nucleotide diversity (*π*) is shown on the upper panel. The schematic diagram shows a black box indicating signal peptide, and the green box is the transmembrane domain. The structural homology domain (head and tail) with PvRBP2b is indicated by amino acid position (aa.). PvRBP1a-N and PvRBP1a-C positions indicate recombinant antigen position. (B) The predicted structure of PvRBP1a (152–625 aa.) showed highly similar structures with PvRBP2b (168–633 aa., PDB ID: 6d03.1) when considering the superimposition between them based on Z-score (38.8) and RMSD (0.6 Å).

The *pvrbp1a* (PVP01_0701200) genomic DNA contains 8,704 bp in length on chromosome 7 with one intron and two exons. The open reading frame contain 8,502 bp (2,833 aa.) in length with 326 kDa predicted molecular weights (Fig 1A). The *pvrbp1a* coding sequence was divided into six segments for detailed characterisation in this study using the following criteria: (1) specific feature domains such as signal peptide and transmembrane domain, (2) homology-based prediction domain which showed binding activity with host cell in other RBLs, (3) polymorphic and conserved sequence. Region I (1–151 aa.) contained signal peptide (1–22 aa.) and unidentified domain with other RBLs. Region II (152–458 aa., head structure) and region III (459–625 aa., tail structure) were divided by protein tertiary structure prediction with PvRBP2b model (PDB ID: 6d03.1) (Fig 1B). Region IV (626–1441 aa.) contained a conserved sequence region and Region V (1442–2771 aa.) was a moderate polymorphic region within *pvrbp1a* sequence from 202 clinical isolates in this study. Region VI (2772–2833 aa.) contained transmembrane (2772–2809 aa.) and intracellular domains (2810–2833 aa.) (Fig 1A).

The protein tertiary structure was predicted by *pvrbp1a* (PVP01_0701200) sequence and the model was best-matched to PvRBP2b binding domain (PDB ID: 6d03.1, 168–633 aa.) (Fig 1A and 1B). The PvRBP1a model Ramachandran plot revealed 91.9% of the favourable regions and 0.4% of disallowed regions in the initial model. The overall quality factor verified by ERRAT showed 91.3%. After refinement, overall structure accuracy was improved to 96.3% of the favourable region and 0.4% of the disallowed region in the Ramachandran plot and to 98.06% of ERRAT. This refined final tertiary structure was allowed as a PvRBP1a prediction model and performed structural alignment with PvRBP2b. The structure was superimposed to 0.6 Å of RMSD and 38.8 of Z-score which reflect that PvRBP1a and PvRBP2b closely resembled (Fig 1B). The PvRBP1a predicted structure model has a clearly divided head (PvRBP1a-RII) and tail (PvRBP1a -RIII) domains based on the PvRBP2b structure which is the reticulocyte binding domain (Fig 1B).

Additionally, PvRBP1a was commonly aligned with conserved hydrophobic regions to other PvRBPs and may have a role in maintaining the proper architecture and specific functions of these proteins [18]. Overall, PvRBP1a-RII and PvRBP1a-RIII were presumed to be the most important domain in eliciting polymorphisms with a conserved conformation.

### Genetic diversity and natural selection of *pvrbp1a*

Across 202 isolates from 6 countries, a total of 139 polymorphic sites, including 31 synonymous and 108 non-synonymous sites, were observed in *pvrbp1a*. A high number of non-synonymous mutation may affect antibody recognition according to changes in the protein tertiary structure. The highest nucleotide diversity was observed in Thailand (*π* ± S.D., 0.00204 ± 0.00007), followed by Ethiopia (0.00171 ± 0.00012), ROK (0.00166 ± 0.00022), Myanmar (0.00159 ± 0.00012), Malaysia (0.00148 ± 0.00011), and China (0.00120 ± 0.00012). However, this result was considered a tendency of high nucleotide diversity according to the sample size which is reflected the gene can be more varied in each country based on sample size. The population-wide nucleotide diversity was 0.00196 ± 0.00004 (*π* ± S.D.) (Table 1). To investigate whether the allele frequencies at the polymorphisms in *pvrbp1a* reflected evidence of selection (departures from random mutation), several tests were performed at both intra- and inter-species levels. Amongst the intra-species tests, Tajima’s D (-1.05156), Fu and Li’s D* (-2.13654, *p* < 0.05), and Fu and Li’s F* (-1.93569, *p* < 0.05) revealed negative values, but only Fu and Li’s D* and F* reached statistical significance (Table 1).

**Table 1:** Estimates of nucleotide diversity, haplotype diversity and neutrality indices of *pvrbp1a-ecto* based on the geographical location.

Location	No. of	SNPs	No. of	Diversity ± S.D.		Tajima’s D	Fu and Li’s	
	samples		haplotype	Haplotype (Hd)	Nucleotide (*π*) X 10^−3^		D*	F*
Thailand	96	82	60	0.987±0.004	2.04±0.07	-0.42848	-0.27082	-0.40383
Malaysia	54	58	11	0.686±0.049	1.48±0.11	-0.22207	-0.85181	-0.73751
Myanmar	10	36	10	1.000±0.045	1.59±0.12	0.19725	-0.21176	-0.12378
China	4	17	4	1.000±0.177	1.20±0.12	0.80053	0.80053	0.82392
ROK	16	47	8	0.892±0.048	1.66±0.22	-0.10865	-0.54111	-0.48353
Ethiopia	22	50	14	0.948±0.029	1.71±0.12	0.14271	0.28773	0.28454
**Overall**	**202**	**139**	**105**	**0.974±0.006**	**1.96±0.04**	**-1.05156**	-2.13654*	-1.93569*

**Fig 2:**
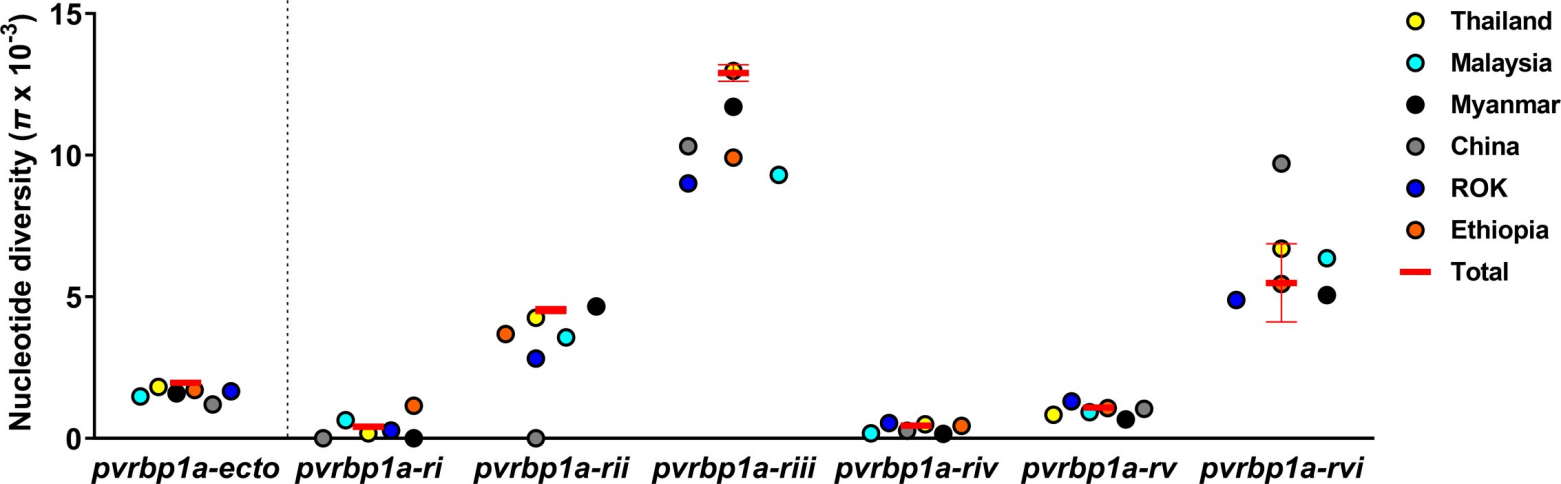
*Pvrbp1a-ecto* and each domain nucleotide diversity (*π*) based on the geographical location. PvRBP1a domain divided into six regions and the structural homolog domain (rii and riii) is highly polymorphic within countries.

On investigation of the six separate *pvrbp1a* coding sequence regions, the highest nucleotide diversity was observed in PvRBP1a-RIII (tail structure, 0.01290 ± 0.00029), followed by PvRBP1a-RII (head structure, 0.00453 ± 0.00012) (Fig 2 and Table 2). Intra-species tests for departures from neutrality for the PvRBP1a-RIII fragment revealed positive values for Tajima’s D (1.13859), Fu and Li’s D* (1.09826), Fu and Li’s F* (1.34179), and dN-dS (1.369) but none reached statistical significance (Tables 2 and 3). Additionally, no significant values were calculated in the inter-species (between *P*. *cynomolgi*) McDonald-Kreitman (MK) test (NI = 1.069) (Table 3). Even all the calculations had weak evidence, all values indicated selection pressure. Overall, the PvRBP1a-RIII domain (tail structure) departure from neutrality was balancing selection that divides the population and contracted within the population.

**Table 2:** Estimates of nucleotide diversity, haplotype diversity and neutrality indices of Pvrbp1a fragments (*p < 0.05, ** p < 0.02, *** p < 0.01).

PvRBP1a region	SNPs	No. of	Diversity ± S.D.		Tajima’s D	Fu and Li’s	
		haplotype	Haplotype (Hd)	Nucleotide (*π*) X 10^−3^		D*	F*
RI	12	12	0.1700±0.0360	0.41±0.09	-2.20689***	-4.16133**	-4.11749**
RII	23	66	0.9540±0.0080	4.53±0.12	0.18145	-0.96778	-0.60847
RIII	27	39	0.9240±0.0090	12.90±0.29	1.13859	1.09829	1.34179
RIV	30	29	0.6180±0.0410	0.45±0.04	-2.21930***	-1.90906	-2.45312*
RV	52	69	0.9580±0.0060	1.09±0.04	-1.51279	-2.14712	-2.24564
RVI	6	7	0.4990±0.0380	4.17±0.43	-0.46163	0.02591	-0.16884
N	53	84	0.9680±0.0060	7.23±0.16	0.56325	0.01404	0.30861
C	15	15	0.4630±0.0420	0.56±2.38	-1.94434*	-1.49092	-1.99727

**Table 3:** McDonald-Kreitman (MK) test on PvRBP1a with PcyRBP1a as out-group species and dN-dS ratio.

PvRBP1a Region (bp)	Polymorphic changes within *P*. *vivax*		Fixed differences between *P*. *vivax* and *P*. *cynomolgi*		Neutrality index (*p* value)^a^	dN-dS (*p* value)
	Syn	Non-syn	Syn	Non-syn		
Overall (1–8499)	31	108	272	743	1.334 (0.170)	1.780 (0.078)
RI (1–453)	4	8	14	34	0.824 (1.000)	-0.660 (0.511)
RII (454–1374)	2	21	45	121	3.905 (0.071)	2.225 (0.028)*
RIII (1375–1875)	2	19	9	80	1.069 (1.000)	1.369 (0.174)
RIV (1876–4323)	8	21	73	191	1.003 (1.000)	-0.240 (0.810)
RV (4324–8313)	15	36	133	316	1.010 (1.000)	-0.545 (0.587)
RVI (8314–8499)	0	6	3	28	-	1.925 (0.057)
N (469–1950)	5	42	55	215	2.149 (0.157)	2.344 (0.021)*
C (1816–2886)	2	13	25	106	1.533 (0.738)	0.803 (0.424)

^a^ Fisher’s exact test *p*-value

There was also no significant evidence of selection at the PvRBP1a-RII fragment with Tajima’s D (0.18145), Fu and Li’s D* (-0.96778), and Fu and Li’s F* (-0.60847) (Table 2). However, the intra-species (within *P*. *vivax*) dN-dS calculation (2.225, *p* < 0.05) indicated significant positive selection (Table 3). The inter-species (between *P*. *cynomolgi*) MK test was greater than 1 in PvRBP1a-RII, indicative of purifying selection (Table 3). Taken together, both PvRBP1a-RII and PvRBP1a-RIII showed high levels of nucleotide diversity that mainly occurred by non-synonymous mutation with weak evidence of balancing selection pressure within species. However, PvRBP1a-RI and PvRBP1a-RIV, which surround the head and tail structure domain, showed significant negative values for Tajima’s D, Fu and Li’s D*, and Fu and Li’s F* indicating that directional selection pressure with population expansion, existing even in low levels of polymorphisms (Table 2). Additionally, the conserved sequence identification was performed by DNAsp software using 202 isolates. The result showed that total of 14 conserved regions were revealed (S2 Table).

**Fig 3:**
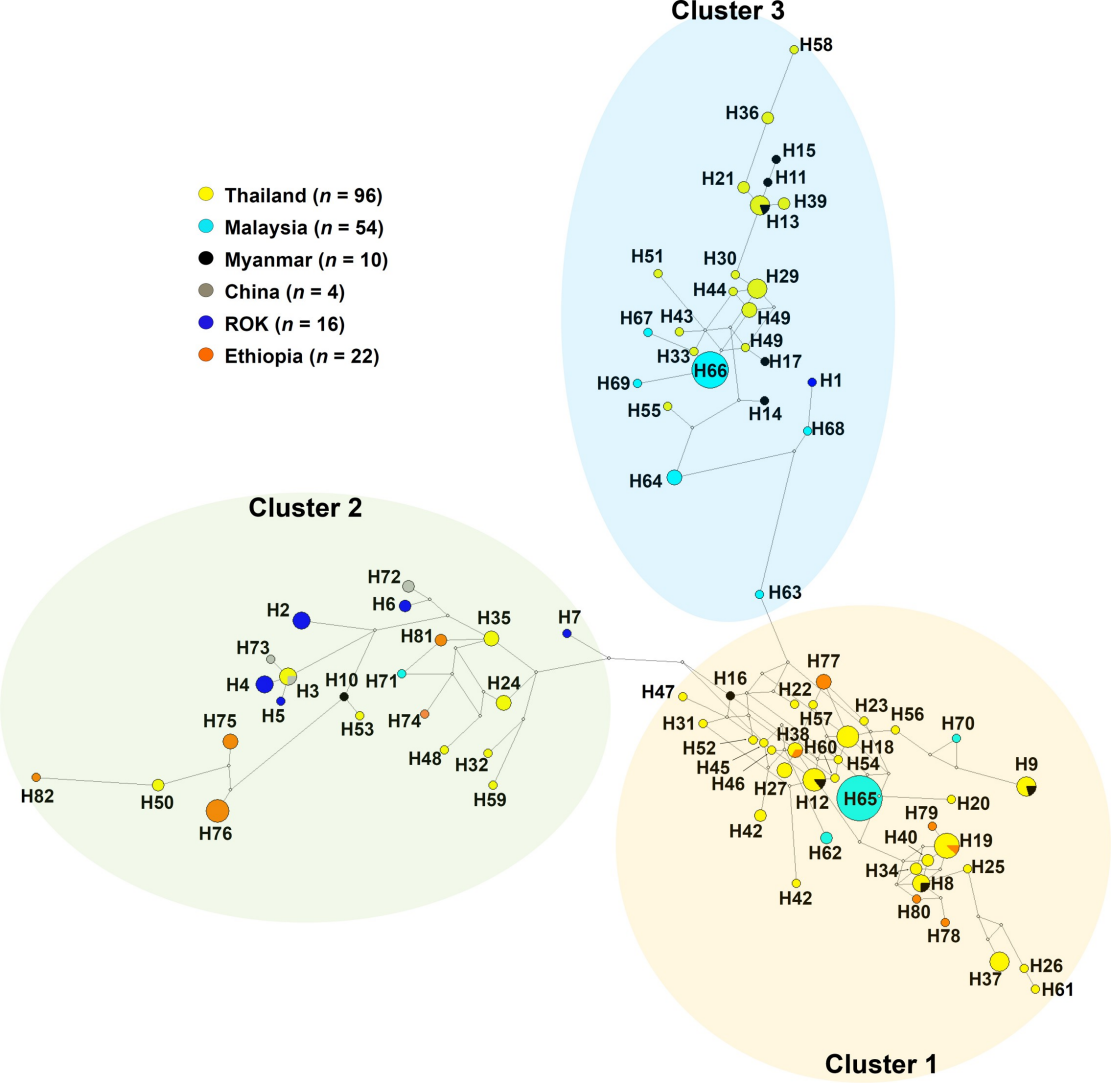
Median-joining networks of structural homolog domain (*pvrbp1a-rii+riii*) haplotype. The geographical haplotype network analysis of *pvrbp1a-rii+riii* region was constructed using the NETWORK 10.2 software with the Median Joining algorithm. The network showed 82 haplotypes found in 202 clinical isolates and was largely divided into three groups.

The haplotype networks using sequence combined across the most polymorphic regions, PvRBP1a-RII+RIII (152–625 aa.), were performed using the Median-joining method. The RII+RIII domains consisted of a total of 82 haplotypes which segregated into three distinct clusters (Fig 3 and S1 Fig). A cluster 1, comprised isolates from across South-East Asia (Malaysia, *n* = 29; Thailand, *n* = 58; Myanmar, *n* = 4) and Africa (Ethiopia, *n* = 8). Cluster 2 appeared to connect with cluster 1 and predominantly comprised isolates from North-East Asia (ROK, *n* = 15; China, *n* = 4) and Africa (Ethiopia, *n* = 14), with little representation from South-East Asia (Malaysia, *n* = 1; Thailand, *n* = 12; Myanmar, *n* = 1). Cluster 3, which also appeared to connect with cluster 1, mainly comprised South-East Asian Asia (Malaysia, *n* = 24; Thailand, *n* = 26; Myanmar, *n* = 5) isolates and one isolate from ROK. The Papua Indonesian PvP01 reference *pvrbp1a* sequence (PVP01_0701200) comprised haplotype 1, located in cluster 3.

### Antigenicity screening

**Fig 4:**
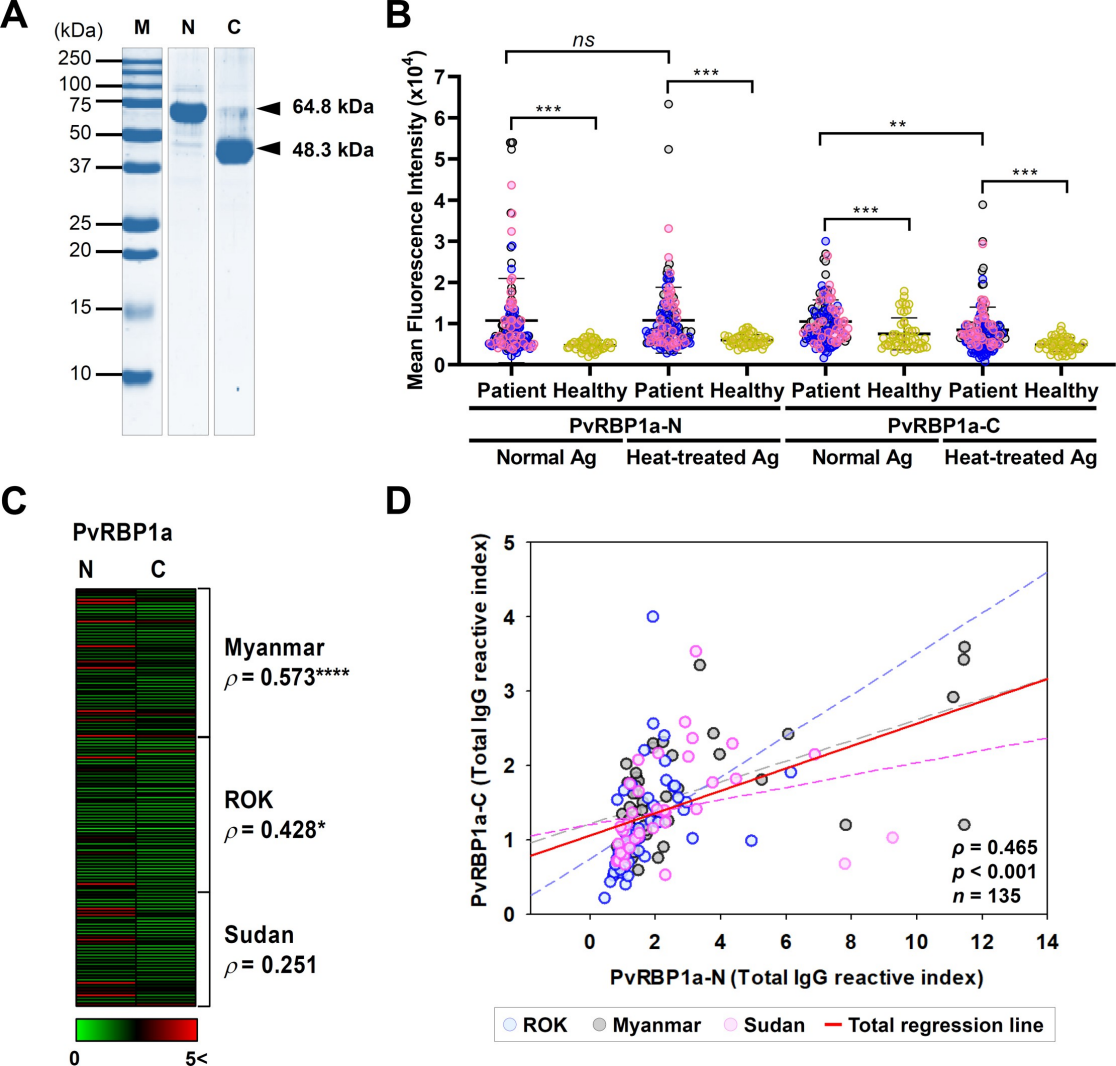
Humoral immune response against PvRBP1a-N and PvRBP1a-C. (A) Purity confirmation by SDS-PAGE of recombinant PvRBP1a-N (157–650 aa.) (64.8 kDa) and PvRBP1a-C (606–962 aa.) (48.3 kDa) expression. (B) Total IgG prevalence of each domain with the vivax patient: Myanmar (grey dot), ROK (blue dot), and Sudan (pink dot) and healthy individual (yellow dot) sera. The bar indicates the mean fluorescence intensity (MFI) ± 95% CI. The *p* values were calculated by Student’s *t*-test. Significant differences are shown as triple asterisks *p* <0.001 and quadruple asterisk *p* <0.0001. (C) IgG prevalence visualized for comparison between N and C with each patient sera by normalized reactivity index. Significant differences are shown as single asterisks *p* <0.05 and quadruple asterisks *p* <0.0001. (D) Correlation between N and C total IgG reactive indices using Pearson correlation test (*ρ*). Blue dot and dash line represent patient sera reactive index from ROK and its regression line. Grey and pink dot and dash line represent reactivity indices and its regression lines from Myanmar and Sudan patient sera, respectively. Red line indicates total regression.

Two PvRBP1a specific domains, PvRBP1a-N (157–650 aa.) (64.8 kDa) and PvRBP1a-C (606–962 aa.) (48.3 kDa), were chosen for antigenicity screening. PvRBP1a-N contains host cell binding domain RII+RIII (head and tail structure), and PvRBP1a-C is a partial sequence of a conserved region in RIV (Fig 1A and 1B). The recombinant proteins were used for the evaluation of antigenicity with three geographically different countries from South-East Asia (Myanmar, *n* = 48), North-East Asia (ROK, *n* = 50), and East Africa (Sudan, *n* = 37) (Fig 4A).

**Fig 5:**
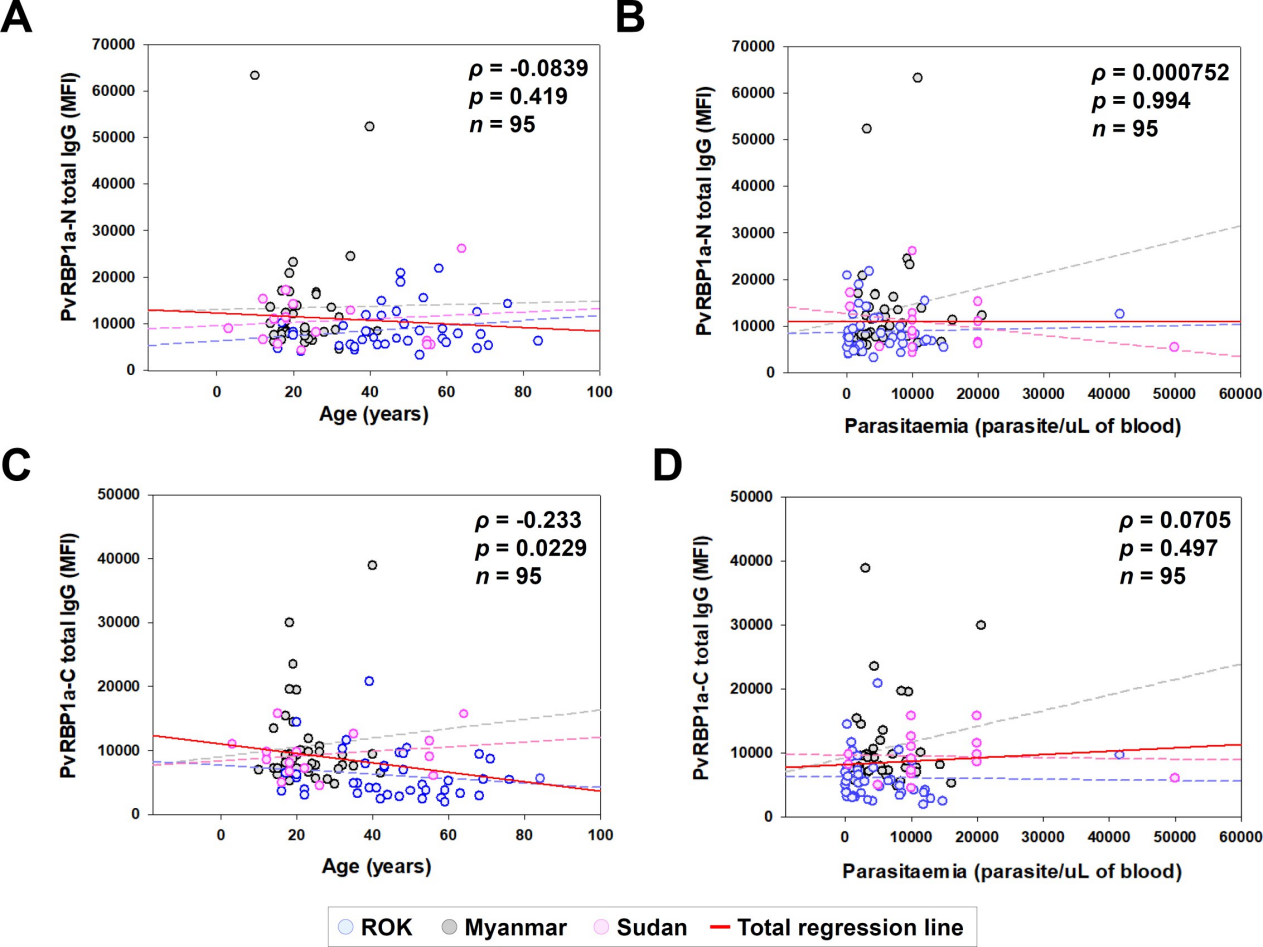
The correlation of parasitaemia and age with PvRBP1a-N and PvRBP1a-C proteins. (A and B) PvRBP1a-N and (C and D) PvRBP1a-C total IgG indices obtained from mean fluorescence intensity (MFI) were evaluated correlation with patient age (years) and parasitaemia (parasite count/uL) by Pearson correlation test (*ρ*), respectively.

Total IgG reactivity in healthy sera was detected in PvRBP1a-N (MFI ± S.D., 4,714 ± 1,251) significantly lower than PvRBP1a-C (7,487 ± 3,881). However, vivax patient sera showed similar in PvRBP1a-N (10,699 ± 10,238) and PvRBP1a-C (10,493 ± 5,269) (Fig 4B). Overall, PvRBP1a specific IgG inducing level after exposure of *P*. *vivax* in PvRBP1a-N (46.7%) was higher than PvRBP1a-C (17.8%) (Table 4). This result indicates that PvRBP1a-N more actively induces antigen-specific IgG by adaptive immune systems than PvRBP1a-C (Fig 4B and Table 4). The epitope characterization was performed by normal and heat-treated proteins antigenicity screening. The comparison between normal and heat-treated antigens showed that PvRBP1a-N was not significantly different, indicating a linear epitope. However, PvRBP1a-C showed a significant difference when structural conformation was destroyed, reflecting a conformational epitope (Fig 4B). The antigenicity correlation analysis between PvRBP1a-N and PvRBP1a-C in individual vivax patient showed positive correlation in Myanmar (*ρ* = 0.573, *p* < 0.001), ROK (*ρ* = 0.428, *p* <0.05), and Sudan (*ρ* = 0.251, *p* = 0.135) (Fig 4C). In the same manner, the overall correlation between PvRBP1a-N and PvRBP1a-C showed a significant positive correlation (*ρ* = 0.465, *p* < 0.001) (Fig 4D). The correlation analysis of PvRBP1a-N and PvRBP1a-C with age and parasitaemia showed that there was no significant correlation except PvRBP1a-C with patient age (*ρ* = -0.233, *p* = 0.0229) (Fig 5A–5D).

**Table 4:** Humoral immune responses against PvRBP1a-N and PvRBP1a-C proteins.

Antigen	No. of patient sample			95% CI^b^	MFI^c^	No. of healthy sample			95% CI	MFI	*p* value^e^
	Pos.	Neg.	Total (%)^a^			Pos.	Neg.	Total (%)^d^			
**PvRBP1a-N (Normal)**	**63**	**72**	**135 (46.7)**	**38.5–55.1**	**10699**	**1**	**49**	**50 (98.0)**	**89.5–99.65**	**4714**	**<0.0001**
Myanmar	26	22	48 (54.2)	40.3–67.4	13343.9						
ROK	19	31	50 (38.0)	25.9–51.9	7695.5						
Sudan	18	19	37 (48.7)	33.5–64.1	11326.3						
**PvRBP1a-N (Heat-treated)**	**61**	**74**	**135 (45.2)**	**37.04–53.6**	**10803**	**2**	**48**	**50 (96.0)**	**86.54–98.9**	**5956**	**<0.0001**
Myanmar	24	24	48 (50.0)	36.39–63.61	12698.85						
ROK	19	31	50 (38.0)	25.86–51.85	9634.331						
Sudan	18	19	37 (48.7)	33.45–64.11	11077.54						
**PvRBP1a-C (Normal)**	**24**	**111**	**135 (17.8)**	**12.3–25.1**	**10493**	**2**	**48**	**50 (96.0)**	**86.54–98.9**	**7487**	**0.0003**
Myanmar	11	37	48 (23.0)	13.3–36.5	12081.9						
ROK	5	45	50 (10.0)	4.4–21.4	8951.6						
Sudan	8	29	37 (21.6)	11.4–37.2	10515.8						
**PvRBP1a-C (Heat-treated)**	**51**	**84**	**135 (37. 8)**	**30.04–46.19**	**8421**	**2**	**48**	**50 (96.0)**	**86.54–98.9**	**4848**	**<0.0001**
Myanmar	22	26	48 (45.8)	32.58–59.71	10333.14						
ROK	12	38	50 (24.0)	14.3–37.41	6070.264						
Sudan	17	20	37 (46.0)	31.04–61.62	9148.775						

^a^ Sensitivity: percentage of positive in patient samples.

^b^ CI: confidence interval.

^c^ MFI: mean fluorescence intensity.

^d^ Specificity: percentage of negative in healthy samples.

^e^ Differences in the total IgG prevalence for each antigen between vivax patients and healthy individuals were calculated with Student *t*-test. A *p* value of < 0.05 is considered statistically significant.

## Discussion

The RBL protein family including PvRBP1a, are essential ligands used by *P*. *vivax* merozoites for the identification and invasion of human reticulocytes, as such they are important potential vaccine candidates [15]. To date studies of PvRBP1a have focused mainly on its reticulocyte binding activity using a limited set of recombinant PvRBP1a fragments (S3 Table) [22,23]. Of particular note, the PvRBP1a recombinant fragment (352–599 aa.) induced antibody, abrogated 40% of *P*. *vivax* merozoite invasion activity [21]. While the results indicate that PvRBP1a is a promising novel vaccine candidate, prior to this study there was limited knowledge about the PvRBP1a genetic diversity and antigen characteristics.

In our study, PvRBP1a-RII and PvRBP1a-RIII (152–625 aa.), located in the N-terminal, were revealed as the most polymorphic regions in the *pvrbp1a* gene. This genetic polymorphism pattern is similar to other RBL protein family members such as PvRBP2a, PvRBP2b, and PfRh5 [18,19,43]. Interestingly, the highly polymorphic PvRBP1a-RII+RIII region overlaps with the RBL domain, showing host cell-binding activity within the RBL family. This domain of PvRBP1a has also been shown to have reticulocyte binding activity (S3 Table) [20,21,23].

Genetic polymorphisms are a major hurdle for vaccine development as variation can alter the epitope expression, resulting in loss of vaccine efficacy [44]. The most advanced *P*. *vivax* vaccine candidate to date is PvDBP-RII (268–513 aa.). The *pvdbp* gene has been show to exhibit high genetic diversity in Malaysia (*π* = 0.01000), Brazil (*π* = 0.01200) [45], and Myanmar (*π* = 0.00400–0.00600), as well as evidence of positive selection pressure [12,46–49]. The PvDBP-RII fragment is a functional domain containing 12 conserved-cysteine residues, with a conserved protein structure and binding functions to DARC [50]. Our study revealed that the PvRBP1a-RII+RIII domains (PvRBP1a-N) have comparable nucleotide diversity (*π* = 0.00748 in worldwide isolates) to PvDBP-RII. Although these domains showed weak evidence for natural selection using intra-species tests, evidence of balancing selection was observed with high level of non-synonymous mutation. However, the inter-species MK test (*NI* = 2.149) was indicated purifying selection. Taken together with the haplotype distribution result, PvRBP1a-RII+RIII is divided into three different genetic clusters. Thus, these three clusters should be considered for PvRBP1a based vaccine development in the future.

Interestingly, the PvRBP1a-RI and PvRBP1a-RIV domains showed strong evidence for directional selection pressure in our study. Both regions exhibited an excess of rare haplotypes (observed once), reflective of a possible population expansion or selection sweep. However, these gene regions (PvRBP1a-C) have low antigenicity, limiting their utility as vaccine candidate targets.

The antigenicity screening results reflect the correlation of PvRBP1a genetic diversity and natural selection properties. PvRBP1a-N (RII+RIII, 157–650 aa.) is a highly polymorphic region mainly caused by random point mutations and could be divided into three genetic clusters. Because the PvRBP1a-N has a linear epitope, conserved epitope sequence residue possibly elicits humoral immune response with up to 46.7% sensitivity. On the other hand, the conformational epitope in PvRBP1a-C (RIV partial, 606–969 aa.) showed lower antigenicity (17.8%) than PvRBP1a-N caused by strong directional selection with population expansion even consist with relatively conserved sequence. In comparison with PvDBP-RII and PvEBP-RII, the same methods for antigenicity screening with this study showed 56.9% and 16.1% sensitivity [23,51]. These data show that PvRBP1a-N elicits moderate level of antigenicity compared with other essential microneme ligands, even it is containing three different genotypes. An effective vaccine should include sequence residues that sufficiently induce the host immune responses and broadly covers the existing antigenic diversity.

To facilitate PvRBP1a based vaccine development, the relationship between sequence conserved regions (S2 Table) and functional studies of PvRBP1a fragment (S3 Table) were compared. Among the PvRBP1a fragments which showed the ability to bind to reticulocytes, the C2 (409–444 aa.) and C3 (473–507 aa.) regions were commonly overlapped, especially the C3 region, which might have important roles to bind reticulocyte when compared to previous report (S3 Table). Furthermore, the antibody against PvRBP1a fragment containing C3 regions showed binding [22] and invasion inhibition activity [20]. Therefore, C3 regions in the PvRBP1a-N (RII+RIII) can be considered attractive target regions for vaccine development.

Although PvRBP1a is a novel candidate for an alternative invasion pathway blocking vaccine, correlation analysis of genetic diversity and antigenicity screening revealed several obstacles. The low antigenicity due to high genetic polymorphism in a particular interest domain, PvRBP1a-RII+RIII will need to be considered for PvRBP1a based vaccine development.

## Supporting information

S1 Table Primer information for sequencing and antigen expression of PvRBP1a. (DOCX)

S2 Table Conserved regions of PvRBP1a-ecto within 202 clinical isolates. (DOCX)

S3 Table Correlation of conserved regions and binding characteristic of PvRBP1a fragments. (DOCX)

S1 Fig Haplotype tree of *pvrbp1a-rii+riii*. The haplotype tree was drawn by the maximum likelihood method and robustness was estimated by the bootstrap method with 1,000 pseudo replicates as implemented in the MEGA7.
(DOCX)
