# Supplementary material for: Geographical distribution and genetic diversity of Plasmodium vivax reticulocyte binding protein 1a correlates with patient antigenicity
Source: PLoS Negl Trop Dis. 2022 Jun 23;16(6):e0010492. doi: 10.1371/journal.pntd.0010492 (PMC9258880; doi:10.1371/journal.pntd.0010492)
Supplement: S1 Table — (DOCX) [file pntd.0010492.s001.docx]

**S1 Table. Primer information for sequencing of PvRBP1a**

| **Gene** | **Primer sequence** |
| --- | --- |
| **Fragment 1** | **F: 5’-CAA CGG CGC AAA TCT ACT AA-3’** |
|  | **R: 5’-TTC TGC ACA TAT TCC CCA CA-3’** |
| **Fragment 2** | **F: 5’-CAG AGA CGT TGG GTG ATA AGG-3’** |
|  | **R: 5’-TCA TAC GAT GCT GTG TTG AGC-3’** |
| **Fragment 3** | **F: 5’-TTA CAG GAT GGG GAA TCT GC-3’** |
|  | **R: 5’-TGA TGT CTT CCC ATT CGT CA-3’** |
| **Fragment 4** | **F: 5’-ACA CAG ATA TTG ATC TTT TGA GG-3’** |
|  | **R: 5’-ATT CCC CAT TGG CTA GCT G-3’** |
| **Fragment 5** | **F: 5’-GAG GGA GAA GGC GAA AAG TC-3’** |
|  | **R: 5’-TCC AGC AAG GCG TTT ATT TC-3’** |
| **Fragment 6** | **F: 5’-GCG GCT AAG GAA AGT TAT GAG-3’** |
|  | **R: 5’-CCG GCT GAT ACG ACT ATT CC-3’** |
| **Fragment 7** | **F: 5’-TCG GAA AAG GTA AGG GAA GC-3’** |
|  | **R: 5’-TTC ATA CTC CGT TTT GGC TTG-3’** |
| **Fragment 8** | **F: 5’-AAC GCT GGA AGA AAT TGA CC-3’** |
|  | **R: 5’-ACC ATC CTG GGT GTT CTC TG-3’** |
| **Fragment 9** | **F: 5’-GAA AAG ACA GCT AGG GCT TT-3’** |
|  | **R: 5’-TGG ACA CCC GTA CGT GTA AG-3’** |
| **PvRBP1a-N** | **F: 5’-ATGTCTGGTGCGGCCGCAATGTACAACAGCATCGTGGA-3’** |
|  | **R: 5’-GAGGTCGACGGCGCGCCGTGCACCTTCTTGGCCAT-3’** |
| **PvRBP1a-C** | **F: 5’-AGTCTTGGAGCGGCCGCAATCTACGAGCTGAAGATCGAGTT-3’** |
|  | **R: 5’-GCTAAGCTTGGCGCGCCGCAGTTGTTGATGGCGC-3’** |
