# Supplementary material for: Geographical distribution and genetic diversity of Plasmodium vivax reticulocyte binding protein 1a correlates with patient antigenicity
Source: PLoS Negl Trop Dis. 2022 Jun 23;16(6):e0010492. doi: 10.1371/journal.pntd.0010492 (PMC9258880; doi:10.1371/journal.pntd.0010492)
Supplement: S2 Table — (DOCX) [file pntd.0010492.s002.docx]

**S2 Table. Conserved regions of PvRBP1a-ecto within 202 clinical isolates.**

| **PvRBP1a fragment** | **Construct**  **name** | **Position** | **Sequence** |
| --- | --- | --- | --- |
|  |  | **Conserved (bp)**  **Covered (aa.)** |  |
| RII | C1 | (641-743 bp)  (215-247 aa.) | AAGTTACAGAATACACAAATTCGTTGAAAACGTTAATGGATTCATGCATATCTGAAAAAGATCAAATGATCATATTAGAGTATGAGATTAATTATGCCAAGAG |
|  | C2 | (1223-1332 bp)  (409-444 aa.) | AAAAATTTGAAGATAATTCCAAAGCGCTAGCAAACAACTACTGCATTTTTCAATACATAAAAACGCTTAACGAACCAATTAAGAAAGCATATGAAAGTAAGGTAATAAAA |
| RIII | C3 | (1415-1522 bp)  (473-507 aa.) | AGTGCAATAAAATTAAAACCGAAGCGGAAAAAGTAAAAGATGACGCTGAAGATATATACGAAAAAAATGAACAGATATACTACGAAATTCCAGAAAGTGAGGACGAAA |
| RIV | C4 | (1980-2136 bp)  (661-712 aa.) | GAAATCAAGTGTGTACTTCACCGAAATGAATGAATTGCTCAACACAGCATCGTATGACAATATGGAGGGGTTTAGCGCTAAGAAGGAGAAGGCGGACAATGACATTAATGCTCTATATAATTCTGTGTATAGGGAAGATATAAATGCTCTCATCGAA |
|  | C5 | (2219-2404 bp)  (741-801 aa.) | ATGCCAAAGAAACTTTTGCTAAATTAAATTTTGTTAGCGATGACAAGCTGACAGATGTGTACACCAAAATGAGTGCAGAAGTGACTAATGCTGAAGGGATCAAAAAAGAAATTGCGCAAAAACAATTCGAAAATGTTCATAAAAAAATGAAAGAATTTTCGGATGCGTTTTCCACAAAATTTGAAG |
|  | C6 | (2586-2750 bp)  (863-916 aa.) | AGGTGAAATATCAGCAGAAATTACCAACATGAGGGAAGTCATAAATAAAATTGAGAGCCAATTAAACTATTACGGGGTTATTGAAAAATATTTCTCCCTCATTGGCGATCAGAATGAAGTGTCAACGGCTAAAGCGTTAAAGGAAAAGATTGTTAGTGATAGTTT |
|  | C7 | (3403-3576 bp)  (1135-1192 aa.) | AATGAAGATTACAAAAAGGTTAAAAATCCAGAAAATGAGAAGCAATTAGAAGCCATAAGGGGATCCATGAGCAAGCTCAAGGAAGTGATCAACAAACACGTCAGCGAAATGACCCAACTGGAGAGCACAGCAAACACTTTAAAGAGCAATGCCAAGGGAAAGGAAAACGAACAC |
|  | C8 | (3696-3919 bp)  (1233-1306 aa.) | AGCAAACAAAGTAGAATTAGAGTTTGAGAGGAACATTATTGGGCACGTTTTAGAACGGATTACAGTAGAAAAGGACAAAGCTGGAAAGGTTGTTGAAGAAATGAATTCTCTCAAAACTAAAATTGAAAAATTGATACAGGAAACAAGTGATGATTCACAAAATGAATTAGTCACAACGAGTATTACAAAACATTTAGAGAATGCAAAGGGGTATGAGGATGTAA |
| RV | C9 | (5292-5609 bp)  (1765-1869 aa.) | TGTTAAAATGAATGAAATTCATGGTGAATTTACCAAATCGTACAATTTGATAGAAACCCATTTGTCCAATGCTACAGATTATTCTGTGACGTTTGAGAAGGCCCAAAGTTTAAGGGAACTAGCAGAGAAGGAAGAAGAACATCTCAGAAGAAGAGAGGAGGAAGCGATCAAACTGCTGAATGATATTAAAAAGGTGGAATCGTTAAAACTGCTAAAAGAAATGATGAAAAAGGTGAGTGCCGAATATGAAGGTATGAAAAGAGACCATACGAGTGTTAGTCAGCTTGTACAGGATATGAAGACAATTGTTGATGAGCT |
|  | C10 | (6555-6759 bp)  (2186-2253 aa.) | TTATGTCGAAACGTTGAAAGGGTTTTACGGTAGTAAGCTTACATTTAGCAGCGCATCCGAAATTGTGCAAAATGCAGACACATATTCCGTGAATTTTGCGAAACACGAAAAGGAGTCTTTAAATGCGATAAGGGATATAAAAAAGGAATTATATTTATTCCACCAAAATAGCGATATTAGCATTGTAGAGGGAGGCGTCCAAAAT |
|  | C11 | (6761-6960 bp)  (2255-2320 aa.) | TGCTGGCACTTTATGATAAGCTGAACGAGGAAAAAAGAGAAATGGATGAACTGTACAGAAATATAAGTGAAACTAAGCTGAAGCAAATGGAACACAGCACTGACGTGTTTAAGCCCATGATAGAATTACACAAAGGAATGAATGAAACGAATAATAAGTCTTTGCTGGAGAAAGAAAAGAAACTAAAAAGCGTGAACGAC |
|  | C12 | (6968-7232 bp)  (2324-2410 aa.) | ACAGTATGGAAGCTGAGATGATTAAAAATGGCCTTAAATACACCCCAGAAAGTGTGCAAAATATTAACAACATATACAGCGTTATTGAAGCTGAGGTGAAAACGCTGGAAGAAATTGACCGTGATTATGGCGATAATTACCAAATCGTGGAAGAGCACAAGAAGCAATTTTCCATTTTAATCGACAGAACGAACGCGCTAATGGATGACATTGAAATTTTTAAAAAGGAAAACAATTACAATTTAATGGAAGTAAACACAGAAAC |
|  | C13 | (7234-7529 bp)  (2412-2509 aa.) | ATACACAGAGTAAACGATTATATAGAAAAGATCACCAATAAGTTAGTACAAGCCAAAACGGAGTATGAACAGATCCTGGAAAATATAAAACAAAACGATGATATGCTACAGAATATTTTTCTCAAAAAAGTAAGTATTATCGAATACTTTGAAAACGTAAAGAAGAAAAAAGAATCTATATTGAACGATTTATATGAACAGGAAAGGCTGCTCAAAATAGGGGAACACTTAGATGAGATTAAGCGTAACGTGACAGAGACACTGAGTAGTTATGAAATTGATCAAAAAATGGAAAT |
|  | C14 | (7960-8241 bp)  (2654-2747 aa.) | GAAATGAGCATGAATAATGATCCCACGCAAAGTGAAACAACTCATTCGGAGGGATCCATCGGTGAAGGAAAAGAATCAGACTCGGATGAAACAGGTTTAACACACGATGCAGGTGCAGATGAAGATTCTACTAGCTCGGCAAAAGGGGCACATGAATTAGAAGAGGAGGAAACTACAGCACCTATGGAAGAAACCGAGATGAATGACAATACCCTTCTAGGGTATGACACTACCAGAAGTGATGAACCTGATATGCATACAGAGAACACCCAGGATGGTACC |
