# Supplementary material for: Geographical distribution and genetic diversity of Plasmodium vivax reticulocyte binding protein 1a correlates with patient antigenicity
Source: PLoS Negl Trop Dis. 2022 Jun 23;16(6):e0010492. doi: 10.1371/journal.pntd.0010492 (PMC9258880; doi:10.1371/journal.pntd.0010492)
Supplement: S3 Table — (DOCX) [file pntd.0010492.s003.docx]

**S3 Table. Correlation of conserved regions and binding characteristic of PvRBP1a fragments.**

| **Region (aa.)** | **Construct** | **Contained conserved  region** | **Binding profile** | | **Inhibition** | | **Ref.** |
| --- | --- | --- | --- | --- | --- | --- | --- |
| 157-481 | F7 | C1, C2 | | No binding | |  | Ntumngia FB et al.,  2018 [1] |
| 157-650 | F8 | C1, C2, C3 | | Reticulocyte (~50 %),  Normocyte (~20 %) | | 100% binding  inhibition by 20 ug/mL of  mouse anti RBP1a-F8 IgG | Ntumngia FB et al.,  2018 [1] |
| 632-976 | F6 | C4, C5, C6 | | Reticulocyte (~20 %), | |  | Ntumngia FB et al.,  2018 [1] |
| 632-1078 | F5 | C4, C5, C6 | | No binding | |  | Ntumngia FB et al.,  2018 [1] |
| 950-1569 | F1 | C7, C8 | | Reticulocyte (~10 %) | |  | Ntumngia FB et al.,  2018 [1] |
| 1542-2192 | F2 | C9 | | Reticulocyte (~20 %) | |  | Ntumngia FB et al.,  2018 [1] |
| 2162-2662 | F3 | C10, C11, C12, C13 | | Reticulocyte (~20 %) | |  | Ntumngia FB et al.,  2018 [1] |
| 351-599 | 34 | C2, C3 | | Reticulocyte (~20 %) | | Binding inhibition by mouse immune sera | Han JH et al.,  2016 [4] |
| 352-599 |  | C2, C3 | | Reticulocyte | | ~ 40 % invasion inhibition of  *P. vivax* by rabbit anti PvRBP1a_30_ antibody | Gupta S et al.,  2018 [2] |
| 160-1170 |  | C1, C2, C3, C4, C5 | | Reticulocyte  Normocyte | |  | Franca CT et al.,  2016 [5] |
| 30-778 | rRBP 1.1 | C1, C2, C3, C4 | | Reticulocyte (~35%) | | ~94% binding inhibition by 800 ug/mL of rat antibody | Gupta ED et al.,  2017 [3] |
| 30-351 | rRBP 1.2 | C1 | | No binding | |  | Gupta ED et al.,  2017 [3] |
| 352-778 | rRBP 1.3 | C1, C2, C3, C4 | | Reticulocyte (~8 %) | |  | Gupta ED et al.,  2017 [3] |
| 352-599 | rRBP 1.4 | C2, C3 | | Reticulocyte (~10 %) | |  | Gupta ED et al.,  2017 [3] |
| 1956-2315 | rRBP1.5 | C10, C11 | | No binding | |  | Gupta ED et al.,  2017 [3] |

**References**

1. Ntumngia FB, Thomson-Luque R, Galusic S, Frato G, Frischmann S, Peabody DS, et al. Identification and Immunological Characterization of the Ligand Domain of *Plasmodium* *vivax* Reticulocyte Binding Protein 1a. The Journal of infectious diseases. 2018;218(7):1110-8. doi: 10.1093/infdis/jiy273. PubMed PMID: 29741629; PubMed Central PMCID: PMC6107737.

2. Gupta S, Singh S, Popovici J, Roesch C, Shakri AR, Guillotte-Blisnick M, et al. Targeting a Reticulocyte Binding Protein and Duffy Binding Protein to Inhibit Reticulocyte Invasion by *Plasmodium vivax*. Scientific reports. 2018;8(1):10511. doi: 10.1038/s41598-018-28757-4. PubMed PMID: 30002416; PubMed Central PMCID: PMC6043553.

3. Gupta ED, Anand G, Singh H, Chaddha K, Bharti PK, Singh N, et al. Naturally Acquired Human Antibodies Against Reticulocyte-Binding Domains of *Plasmodium vivax* Proteins, PvRBP2c and PvRBP1a, Exhibit Binding-Inhibitory Activity. The Journal of infectious diseases. 2017;215(10):1558-68. doi: 10.1093/infdis/jix170. PubMed PMID: 28379500; PubMed Central PMCID: PMC5853946.

4. Han JH, Lee SK, Wang B, Muh F, Nyunt MH, Na S, et al. Identification of a reticulocyte-specific binding domain of *Plasmodium vivax* reticulocyte-binding protein 1 that is homologous to the PfRh4 erythrocyte-binding domain. Scientific reports. 2016;6:26993. doi: 10.1038/srep26993. PubMed PMID: 27244695; PubMed Central PMCID: PMC4886630.

5. Franca CT, He WQ, Gruszczyk J, Lim NT, Lin E, Kiniboro B, et al. *Plasmodium vivax* Reticulocyte Binding Proteins Are Key Targets of Naturally Acquired Immunity in Young Papua New Guinean Children. PLoS neglected tropical diseases. 2016;10(9):e0005014. doi: 10.1371/journal.pntd.0005014. PubMed PMID: 27677183; PubMed Central PMCID: PMC5038947.
