# Supplementary material for: Geographical distribution and genetic diversity of Plasmodium vivax reticulocyte binding protein 1a correlates with patient antigenicity
Source: PLoS Negl Trop Dis. 2022 Jun 23;16(6):e0010492. doi: 10.1371/journal.pntd.0010492 (PMC9258880; doi:10.1371/journal.pntd.0010492)
Supplement: S1 Fig — The haplotype tree was drawn by the maximum likelihood method and robustness was estimated by the bootstrap method with 1,000 pseudo replicates as implemented in the MEGA7. (DOCX) [file pntd.0010492.s004.docx]

**

**

**S1 Fig. Haplotype tree of *pvrbp1a-rii+riii*.** the haplotype tree was drawn by the maximum likelihood method and robustness was estimated by the bootstrap method with 1,000 pseudo replicates as implemented in the MEGA7.
